# Acute Changes in Liver Function Tests During Initiation of Ketogenic Diet

**DOI:** 10.1177/08830738241272063

**Published:** 2024-09-16

**Authors:** Akshat Katyayan, Anuranjita Nayak, Gloria Diaz-Medina, Maureen Handoko, James John Riviello

**Affiliations:** Department of Pediatric Neurology and Developmental Neuroscience, Texas Children's Hospital, 3989 Baylor College of Medicine, Houston, TX, USA

**Keywords:** epilepsy, hepatotoxicity, ketogenic diet, liver function tests

## Abstract

**Background:**

Ketogenic diet is an effective therapy for patients with medically refractory epilepsy. It is generally well tolerated, with the most common side effects being gastrointestinal. Hepatic toxicity has been described as an uncommon side effect of ketogenic diet, usually with long-term use. However, there are limited data to implicate ketogenic diet in acute liver toxicity.

**Methods and results:**

We analyzed all patients who underwent elective inpatient ketogenic diet initiation at our institution from June 2019 to June 2022. Of the 25 patients reviewed, we found 6 patients who showed acute, asymptomatic changes in liver function tests during initiation, in both hepatocellular and cholestatic patterns. Two patients stopped the ketogenic diet acutely and 3 patients continued ketogenic diet with changes in medications and/or addition of choline—all patients had improvement and normalization of liver function tests in the short term. One patient had acute normalization of chronically elevated liver function tests on ketogenic diet initiation.

**Conclusion:**

Ketogenic diet can cause acute changes in liver function tests during initiation of ketogenic diet, with both hepatocellular and cholestatic patterns, with and without the concurrent use of hepatotoxic medications. In most patients, ketogenic diet can be continued successfully by making changes to medications or addition of choline.

Ketogenic diet is a high-fat, adequate-protein, and low-carbohydrate dietary therapy that has long been used in the management of patients with refractory epilepsy. It is the treatment of choice for glucose transporter 1 deficiency syndrome (GLUT1DS) and pyruvate dehydrogenase deficiency (PDHD) but is also particularly beneficial in patients with Dravet syndrome, epileptic spasms, and other epileptic encephalopathies.^1^

The side effects of ketogenic diet are well described and commonly include acute gastrointestinal side effects (emesis, constipation, and abdominal pain), hypoglycemia, and acidosis. Chronic side effects include hyperlipidemia, renal calculi, growth deceleration, and pancreatitis, among others.^1^ Hepatotoxicity has been described in up to 5.7% of patients on ketogenic diet and includes elevation of liver enzymes and liver steatosis.^2^ Several other case reports have described either long-term hepatotoxicity with ketogenic diet^3^ or short-term hepatotoxicity confounded by use of hepatotoxic medications and illness,^4^ but there are no clear data documenting acute hepatic function changes during initiation of ketogenic diet.

The recommendations of the International Ketogenic Diet Study Group is to check serum β-hydroxybutyrate and serum glucose during the initiation of ketogenic diet^1^; however, checking liver function tests is not recommended. This case series provides a descriptive analysis of all patients who showed acute changes in liver function tests during inpatient initiation of ketogenic diet at Texas Children's Hospital.

## Methods

All patients who underwent inpatient ketogenic diet initiation from June 2019 to June 2022 were analyzed. Emergency ketogenic diet initiations in the ICU and outpatient initiations were excluded. It is standard practice at our center to check daily serum β-hydroxybutyrate and a comprehensive metabolic panel (including electrolytes and liver enzymes) during ketogenic diet initiation.

A total of 25 patients underwent elective inpatient initiation, with successive, daily increase in the ketogenic diet ratio depending on laboratory test results. Of those, we identified 6 patients who showed evidence of acute changes in liver function tests during initiation. All patients were screened prior to initiation of ketogenic diet with standard preinitiation laboratory tests, including total and free carnitine, acyl carnitine and urine acyl glycine profile, comprehensive metabolic panel, vitamin D, plasma amino acids, urine organic acids and fasting lipid panel, urine calcium and creatinine and a baseline EKG. All patients had screening laboratory tests within broad limits of normal, except when otherwise noted. All patients who were continued on ketogenic diet achieved adequate ketosis, defined as a serum β-hydroxybutyrate level of 2 mmol/L or greater.

The reference values for liver enzymes at the laboratory at Texas Children's Hospital are as follows: alkaline phosphatase, 104-345 U/L; aspartate aminotransferase, 3-56 U/L; and alanine aminotransferase, 5-30 U/L. In each patient, specific liver function tests were abnormal, as noted. In all patients, serum albumin and bilirubin remained normal.

Table 1 summarizes the pertinent details of all 6 patients.

**Table 1.** Summarizing Patient Information of the 6 Patients.

Patient number	Age at KD initiation	Etiology	Use of concurrent hepatotoxic medications	Pre-initiation LFT	Type of acute LFT changes	Intervention
1	2 y	Genetic (SCN1A mutation- Dravet syndrome)	Yes (VPA, clobazam, cannabidiol)	Normal	Hepatocellular-AST and ALT elevated 6-8 times compared to baseline	KD stopped
2	12 mo	Structural (perinatal hemorrhage)	No	Normal	Hepatocellular-AST and ALT elevated 8-13 times compared to baseline	KD stopped
3	5 y	Genetic (adenylosuccinate lyase deficiency)	Yes (cannabidiol, clobazam)	Normal	Hepatocellular-AST and ALT elevated 6-18 times compared to baseline	KD continued at 2:1 ratio, clobazam weaned off
4	15 mo	Structural (left MCA stroke)	Yes (bosentan)	Mildly elevated	Hepatocellular-AST and ALT elevated 1.5-2 times compared to baseline	KD continued at 1.5:1 ratio, bosentan stopped
5	10 mo	Structural (bilateral intraventricular hemorrhage)	Yes (clobazam)	Normal	Cholestatic (ALP elevated 16 times normal compared to baseline)	KD continued at 2:1 ratio, choline supplementation added
6	3 y	Genetic (SCN2A early infantile epileptic encephalopathy)	Yes (phenytoin, phenobarbital)	Elevated (mixed pattern)	LFTs normalized	KD continued at 2.75:1 ratio, choline supplementation added

Abbreviations: ALP, alkaline phosphatase; ALT, alanine aminotransferase; AST, aspartate aminotransferase; KD, ketogenic diet; LFT, liver function test; MCA, middle cerebral artery; VPA, valproic acid.

## Patient Descriptions

### Patient 1

The patient was a 2-year-old boy with Dravet syndrome due to de novo mutation in the *SCN1A* gene. The patient was diagnosed with seizures at 4 months of age, when he presented with prolonged right hemibody convulsions in the setting of febrile illness. The patient continued to have multiple episodes of hemibody (mostly right, sometimes left) convulsive status epilepticus. At around 1 year of age, he developed other unprovoked seizure types, including atonic and atypical absence seizures. This was associated with developmental regression.

The patient was hence referred to the ketogenic diet clinic for consideration of dietary therapies.

Preinitiation ketogenic diet laboratory test results were normal. Comprehensive metabolic panel was also unrevealing, with normal alkaline phosphatase (234 U/L), aspartate aminotransferase (41 U/L), and alanine aminotransferase (31 U/L). At that time, the patient was on clobazam (1 mg/kg/d), valproic acid (27 mg/kg/d), and cannabidiol (Epidiolex; 17 mg/kg/d).

**Figure 1.**
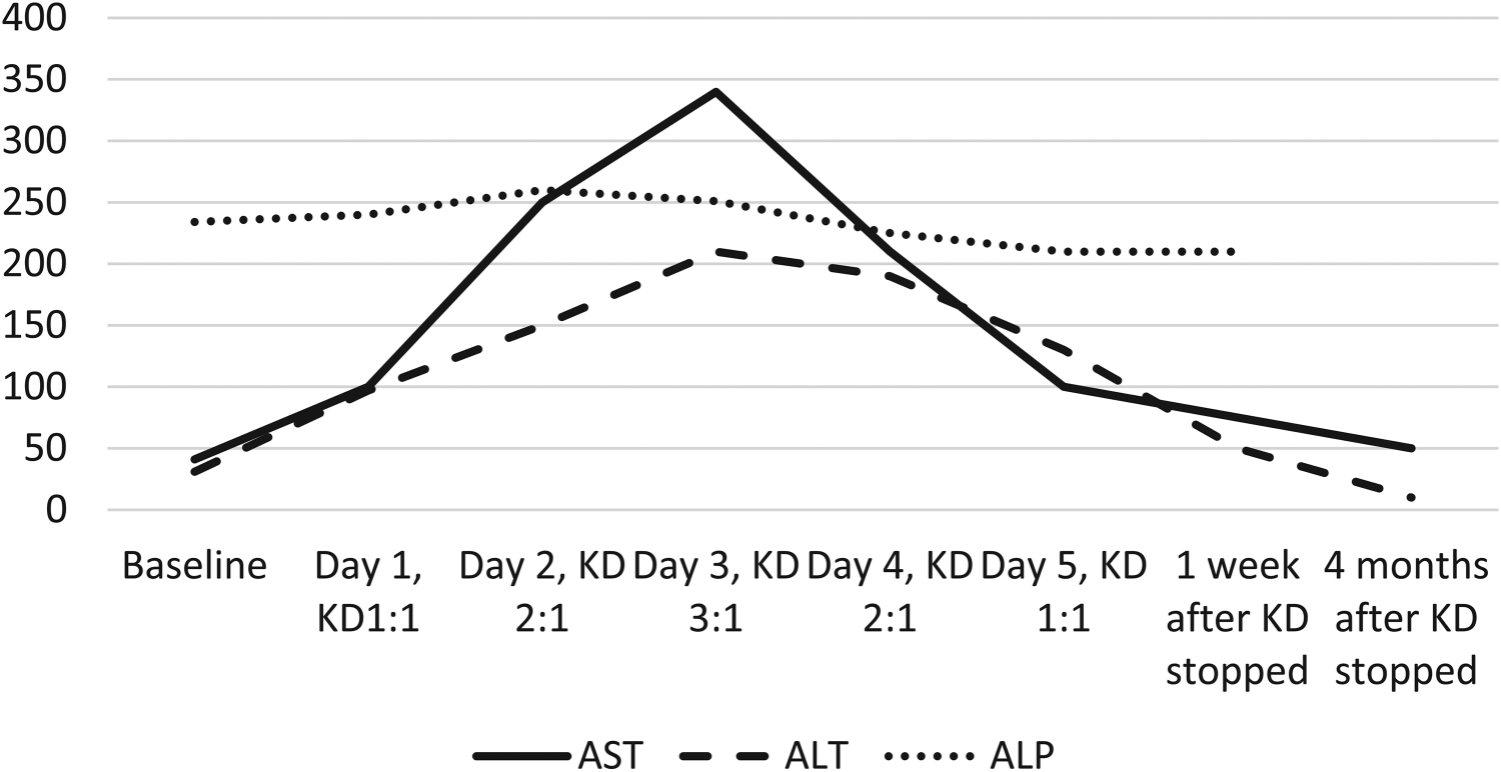
Graph showing elevation of alanine aminotransferase (ALT) and aspartate aminotransferase (AST) with daily increase in ketogenic diet ratio, patient 1.

During inpatient ketogenic diet initiation 2 weeks later, serial comprehensive metabolic panels and blood ketone levels were obtained. Liver enzymes, both aspartate aminotransferase and alanine aminotransferase, showed significant elevation (Figure 1), corresponding to successive increases in the ratio of ketogenic diet, with aspartate aminotransferase showing an 8-fold elevation at the highest ratio of 3:1 and alanine aminotransferase showing a corresponding 6-fold elevation. Alkaline phosphatase remained stable. Patient remained asymptomatic and tolerated the ketogenic diet well, with no notable side effects. He achieved mild ketosis with the highest β-hydroxybutyrate level of 1.1 mmol/L on the 3:1 ratio, with no significant hypoglycemia or acidosis. Per family, all antiseizure medications were effective in reducing seizures, and because of concern for breakthrough status epilepticus during weaning of antiseizure medications, the ketogenic diet was weaned off over 3 days and the liver enzymes showed a corresponding reduction acutely, with no changes made to antiseizure medications.

### Patient 2

The patient was a 12-month-old girl, who was born at 30 weeks of gestation due to preeclampsia. She was found to have multiple bilateral intraparenchymal hemorrhagic lesions resulting in neonatal seizures, which subsequently evolved to multifocal cystic encephalomalacia and ex vacuo hydrocephalus, with resultant global developmental delay and cortical visual impairment.

At around 5 months of age, she developed epileptic spasms and tonic seizures of varying and asymmetric semiology, occurring in clusters and isolation. She failed multiple antiseizure medications, including phenobarbital, oral prednisolone, zonisamide, clobazam, and topiramate. At the time of evaluation in the ketogenic diet clinic, the family had stopped all antiseizure medications except for topiramate at 1 mg/kg/d because they felt that medications resulted in side effects and did not help with seizures.

Preinitiation ketogenic diet laboratory test results were normal. Specifically, baseline comprehensive metabolic panel obtained 2 months prior to the visit showed normal alkaline phosphatase (167 U/L), aspartate aminotransferase (18 U/L), and alanine aminotransferase (22 U/L).

**Figure 2.**
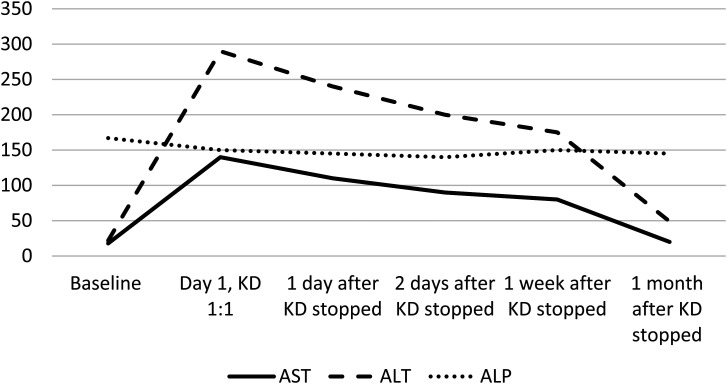
Graph showing elevation of alanine aminotransferase (ALT) and aspartate aminotransferase (AST) with daily increase in ketogenic diet ratio, patient 2.

Ketogenic diet was started inpatient and the same laboratory testing was performed. As with prior patient, both aspartate aminotransferase and alanine aminotransferase showed significant elevation (Figure 2), after starting the diet at 1:1 ratio, with aspartate aminotransferase showing an 8-fold increase and alanine aminotransferase showing a 13-fold increase the day after introduction of the ketogenic diet. Alkaline phosphatase levels continued to be normal. Because of significant elevation in transaminases, ketogenic diet was stopped on day 2 and transaminases started showing a downward trend during the hospitalization and returned to normal within a month of discharge.

Because this patient had no concomitant use of significant hepatotoxic medications, a genetics referral was made to evaluate for potential metabolic defects, which may explain acute asymptomatic hepatocellular toxicity with the ketogenic diet. Whole exome sequencing and comprehensive mitochondrial DNA analysis by massively parallel sequencing (mt-NGS) were unrevealing and hence no alternate explanation was found.

### Patient 3

The patient was a 5-year-old girl with medically refractory epilepsy, developmental delay, and vision deficits associated with adenylosuccinate lyase deficiency (ADSL) with a homozygous pathogenic variant (c.1277G>A) in the *ADSL* gene. She had a history of epileptic spasms during infancy, which resolved with short-term vigabatrin treatment. She continued to have seizures that were intermittently well controlled until around 4 years of age when she again had worsening of tonic seizures, at times progressing to generalized tonic-clonic activity.

At the time of initial evaluation for ketogenic diet therapy, she was exclusively gastrostomy (G)-tube dependent for her nutrition. Pre–ketogenic diet initiation laboratory test results were normal. Specifically, baseline comprehensive metabolic panel obtained 7 weeks prior to the initiation showed normal alkaline phosphatase (158 U/L), aspartate aminotransferase (41 U/L), and alanine aminotransferase (17 U/L).

**Figure 3.**
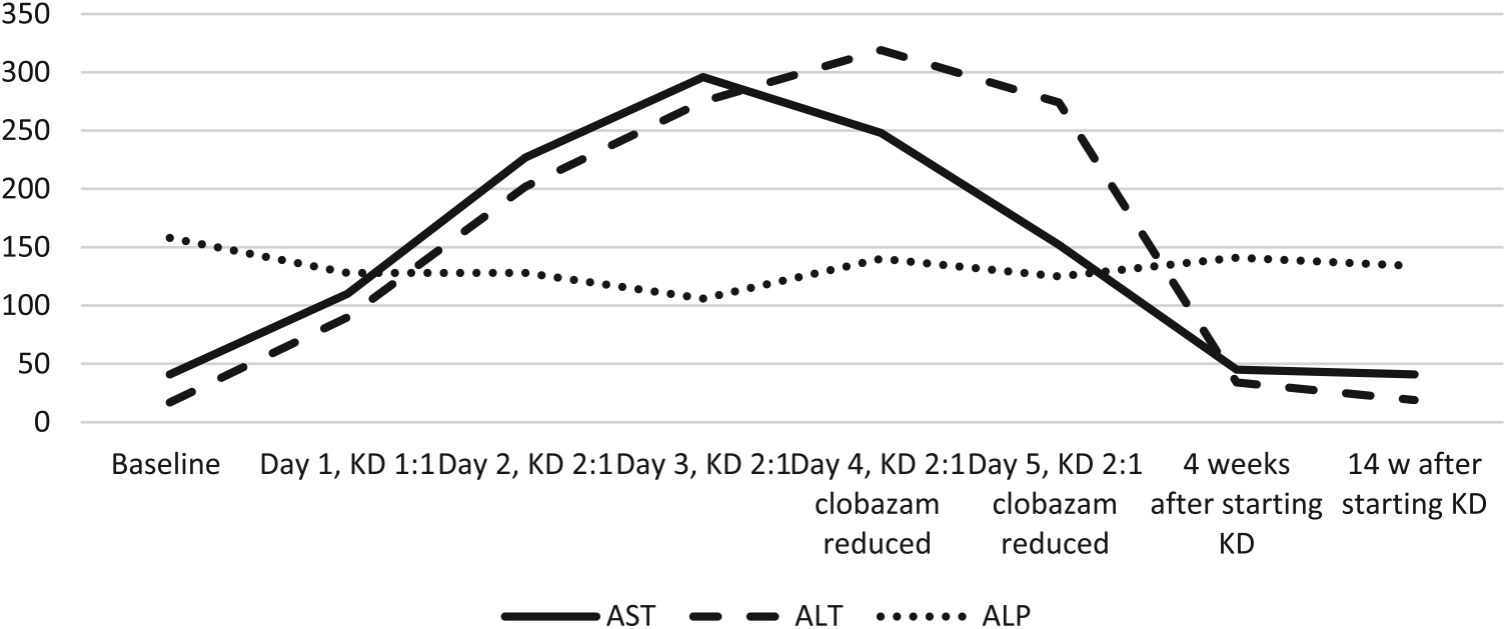
Graph showing elevation of alanine aminotransferase (ALT) and aspartate aminotransferase (AST) with successive increase in ratio of ketogenic diet and later normalization, patient 3.

At the time of admission for ketogenic diet initiation, she was on cannabidiol (25 mg/kg/d), clobazam (0.8 mg/kg/d), levetiracetam (55 mg/kg/d), and rufinamide (22 mg/kg/d). Clobazam dose had been decreased because of side effects of hypotonia and worsening of seizures, and rufinamide had been added 1 month before admission. On starting the ketogenic diet, she was found to have elevated aspartate aminotransferase (110 U/L) and alanine aminotransferase (90 U/L). Over the subsequent 3 days, her aspartate aminotransferase and alanine aminotransferase continued to increase but appeared to be plateauing with peak values of aspartate aminotransferase 248 U/L and alanine aminotransferase 319 U/L on day 4 of admission, at the highest ketogenic diet ratio of 2:1, which she otherwise tolerated without difficulty. Clobazam wean was started at this time due to lack of efficacy and to simplify medication regimen as polypharmacy being a contributor to transaminitis. On day 5 of admission, transaminases had trended down to alanine aminotransferase of 274 U/L and aspartate aminotransferase of 152 U/L. Because liver function tests had stabilized, the decision was made to continue ketogenic diet. One month after discharge, alanine aminotransferase decreased to 34 U/L and aspartate aminotransferase to 45 U/L. Three months after discharge, liver function tests continued to be normal, and ketogenic diet was continued with >50% reduction in seizures. The liver function test trend is shown in Figure 3.

### Patient 4

The patient was a 15-month-old boy born at full term with pulmonary hypertension and respiratory failure secondary to a variant in CTNNB1 gene. He required extracorporeal membrane oxygenation following cardiac arrest during which he sustained a left middle cerebral artery stroke, which resulted in right hemiparesis. At 11 months of age, he developed epileptic spasms consisting of clusters of eye rolling movements with stiffening of bilateral extremities. He was initially started on high-dose prednisolone and later switched to vigabatrin, which was poorly tolerated and hence was weaned off. The family initially refused epilepsy surgery; hence he was evaluated for ketogenic diet.

**Figure 4.**
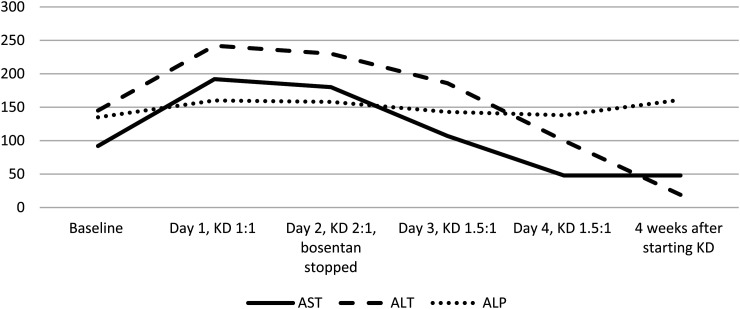
Graph showing elevation of alanine aminotransferase (ALT) and aspartate aminotransferase (AST) with successive increase in ratio of ketogenic diet and later normalization, patient 4.

Preinitiation ketogenic diet laboratory test results were normal, except for baseline elevation of aspartate aminotransferase (92 U/L) and alanine aminotransferase (145 U/L) with normal alkaline phosphatase (135 U/L). This was thought to be related to Bosentan for pulmonary hypertension and was being monitored. On admission for ketogenic diet, the patient was on levetiracetam 35 mg/kg/d and clonazepam 0.03 mg/kg/d. On starting ketogenic diet at 1:1 ratio, liver enzymes showed acute elevation, with aspartate aminotransferase increasing to 192 U/L and alanine aminotransferase increasing to 242 U/L. This prompted discussion with the cardiology team, which elected to stop the Bosentan. Liver function tests were monitored successively and returned to normal, as is shown in Figure 4.

The patient showed a good response to ketogenic diet and tolerated it well, with a 70% to 80% reduction in epileptic spasms at the 3-month mark. However, spasms increased 6 months after ketogenic diet initiation and at that time, the family agreed for a left functional hemispherectomy. The patient became seizure free after the surgery and ketogenic diet was weaned off 1 year after the surgery. His liver function tests continued to be normal while he remained on ketogenic diet.

### Patient 5

The patient was a 10-month-old male born at 36 weeks of gestation with perinatal bilateral (right greater than left) intraventricular hemorrhage with hydrocephalus, requiring placement of a ventriculoperitoneal shunt. Patient developed focal impaired aware seizures in the neonatal period described as eye deviation to the left with behavioral arrest of right posterior temporal onset. The patient was initially on phenobarbital and levetiracetam, and later clobazam was added. Patient developed mixed flexor-extensor spasms of generalized onset at 4 months of age. Patient failed prednisolone and vigabatrin and hence was started on ketogenic diet.

Pre–ketogenic diet initiation laboratory test results were normal. Specifically, baseline comprehensive metabolic panel obtained 1 month prior to the initiation showed normal alkaline phosphatase (137 U/L), aspartate aminotransferase (60 U/L) and alanine aminotransferase (29 U/L). At the time of initiation, the patient was on levetiracetam at 60 mg/kg/d, clobazam at 1 mg/kg/d, and vigabatrin at 150 mg/kg/d.

**Figure 5.**
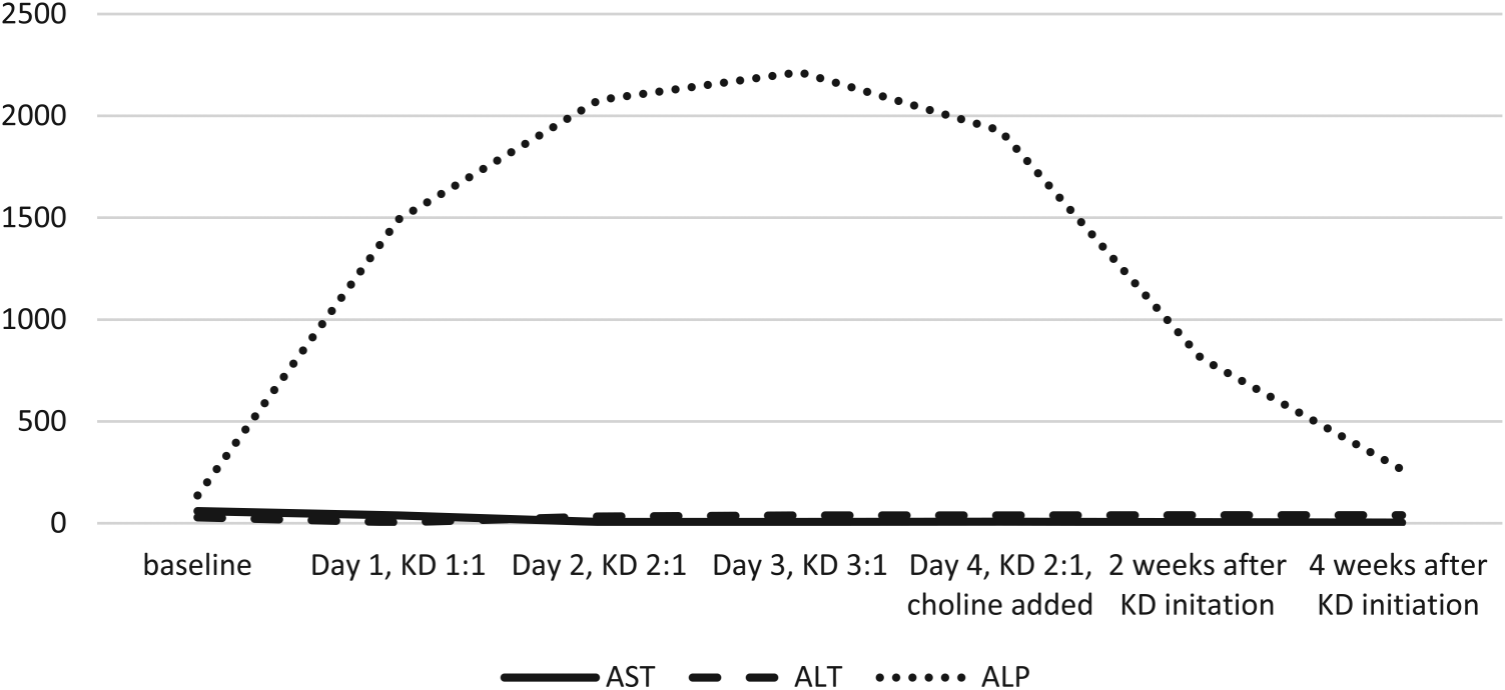
Graph showing elevation of alkaline phosphatase with successive increase in ratio of ketogenic diet: patient 5.

On initiation of ketogenic diet at 1:1 ratio, the patient showed acute elevation of alkaline phosphatase up to 16 times normal compared with baseline (from 137 U/L to 1491, 2078, and 2215 U/L on days 1, 2, and 3, respectively). Other liver function tests, including aspartate aminotransferase and alanine aminotransferase, continued to be normal. Patient tolerated ketogenic diet initiation well and achieved adequate ketosis. After consultation with endocrinology and gastroenterology services, other laboratory tests to rule out liver and bone dysfunction were ordered, including γ-glutamyl-transferase, phosphorus, parathormone, serum human chorionic gonadotropin and 25-hydroxyvitamin D, which were all normal. Patient was started on choline (sunflower lecithin at 2.5 g/kg) on day 3 of initiation, and alkaline phosphatase levels gradually returned to normal after 4 weeks of initiation, as is shown in Figure 5. The patient continues on ketogenic diet with >50% reduction in seizures with normal alkaline phosphatase levels.

### Patient 6

The patient was a 3-year-old girl with a history of SCN2A-related early infantile epileptic encephalopathy, with generalized brain volume loss, paucity of deep cerebral white matter, and thinning of corpus callosum and brainstem structures. Patient had multiple daily seizures of varying semiology, including asymmetric tonic, evolving into bilateral convulsive seizures, focal impaired aware with initial left eye deviation, evolving into bilateral convulsive seizures and frequent atypical absence seizures. The patient needed multiple rescue medications at home and required frequent emergency department visits and hospital admissions. The patient had global developmental delay at baseline and was tracheostomy and gastrostomy tube dependent.

She had failed multiple antiseizure medications but had shown the best response to sodium channel blockers.

At the time of initial evaluation in the ketogenic diet clinic, her preinitiation laboratory test results were normal, except for chronic elevation in her liver function tests. At baseline, 3 months prior to ketogenic diet initiation, her aspartate aminotransferase was 151 U/L, alanine aminotransferase was 299 U/L and her alkaline phosphatase was normal at 177 U/L. γ-Glutamyl-transferase was elevated as well, at 1226 U/L. Patient has been extensively evaluated by gastroenterology, and the cause of chronic elevation in liver enzymes was thought to be due to antiseizure medications, which included phenytoin at 3 mg/kg/d, oxcarbazepine at 60 mg/kg/d, phenobarbital at 4 mg/kg/d, and also lacosamide at 10 mg/kg/d and zonisamide at 12 mg/kg/d.

**Figure 6.**
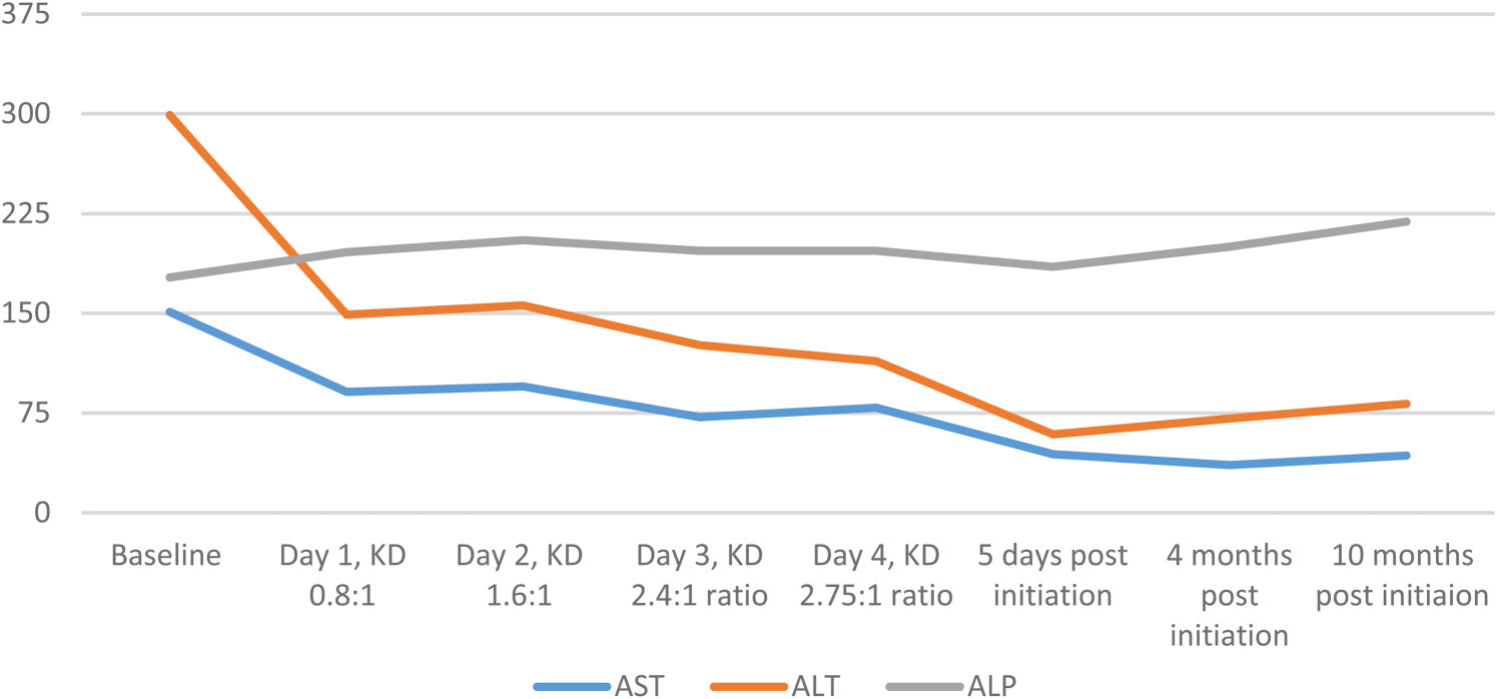
Graph showing downtrending of alanine aminotransferase (ALT) and aspartate aminotransferase (AST) with successive increase in ratio of ketogenic diet + choline: patient 6.

The patient was cautiously started on ketogenic diet with choline (sunflower lecithin at 4.5 g/kg) and ratio was increased successively. She achieved adequate ketosis. Her liver function tests, specifically her aspartate aminotransferase and alanine aminotransferase, showed acute improvement even during ketogenic diet initiation, with some minor fluctuations (Figure 6). Her γ-glutamyl-transferase levels also showed a reduction, compared with the baseline of 1226 U/L, but remained elevated between 646 and 851 U/L, with random fluctuations during the initiation. Patient tolerated ketogenic diet initiation well. Three months after initiation, aspartate aminotransferase and alanine aminotransferase continued to be significantly improved compared with the pre–ketogenic diet baseline, a trend that continued long-term. The patient achieved greater than 80% of seizure reduction in the long term. γ-Glutamyl-transferase continued to show modest elevation, but no interventions have been recommended by the gastroenterologist.

## Discussion

Liver function tests typically include alanine aminotransferase, aspartate aminotransferase, alkaline phosphatase, γ-glutamyl-transferase, serum bilirubin, and albumin, among others. Abnormal liver function tests can be classified into 2 broad patterns: hepatocellular pattern (disproportionate elevation in aspartate aminotransferase and alanine aminotransferase) and cholestatic pattern (disproportionate elevation in alkaline phosphatase, γ-glutamyl-transferase, and bilirubin).^5^

Although studies have shown elevated liver enzymes in patients on ketogenic diet long term,^2^ to our knowledge, there is no definitive data directly implicating ketogenic diet in acute derangements in liver function tests during initiation.

One case report^4^ described an 18-month-old girl who showed a remarkable elevation in liver enzymes 2 days after discharge. However, this patient was also on valproic acid valproic acid and had a concurrent febrile illness and tolerated the ketogenic diet later once the illness resolved and valproic acid was discontinued. Other studies^2,6^ have shown elevated transaminases in a minority (2.1% and 2.3% respectively) of patients on ketogenic diet only after 1 month of initiation of therapy.

Patient 1 was on a combination of valproic acid, clobazam, and cannabidiol. All these antiseizure medications individually and in combination^7^ have been shown to elevate liver enzymes. Because this combination had resulted in significant improvement of seizures and past attempts at withdrawal of medications had resulted in status epilepticus, the team felt it was safer to continue the current combination of medications and reintroduce ketogenic diet later if valproic acid was able to be weaned off.

Ketogenic diet and valproic acid both have been known to cause chronic hepatotoxicity independently. There have been publications both in favor of and against the concomitant use of these two agents.^3,8^

The mechanism of this hepatoxicity is not clearly known. There are several proposed hypotheses:

1. Elevated plasma concentration of fatty acids are seen in patients on ketogenic diet and this may compete with valproic acid (which itself is a fatty acid) for binding sites leading to an increase in valproic acid levels.^9^
2. Both ketogenic diet and valproic acid can cause liver injury through causing carnitine deficiency, which is important in long-chain fatty acid metabolism.^9^
3. Inhibition of mitochondrial beta oxidative phosphorylation by valproic acid,^3^ especially in choline-deficient states.^2^
4. Chronic ketogenic diet usage causes an increase in hepatocyte inflammatory markers through various mechanisms; however, if the same holds true in the acute state is unknown.

However, in patient 2, no other clear cause of hepatoxicity was found except for being on very low dose of topiramate, which was reported to be hepatotoxic in a single case report^10^ but otherwise very uncommon. Confounding genetic and metabolic factors were excluded by negative whole exome and mitochondrial DNA sequencing and hence the side effects can only be attributable to ketogenic diet and are further supported by serial reduction of liver enzymes as the ketogenic diet was stopped (Figure 2) without making any changes to other medications.

Similarly, patients 3 and 4 were on medications known to affect liver function tests (clobazam and bosentan) and liver enzymes, which acutely increased after the start of ketogenic diet showed improvement as the medications were withdrawn.

However, patient 5 showed a cholestatic pattern of liver function test abnormality (elevated alkaline phosphatase, however, with normal γ-glutamyl-transferase and bilirubin, and normal aspartate aminotransferase and alanine aminotransferase). Alkaline phosphatase abnormalities are not exclusive to liver disease and can also be seen in bone disease, which were ruled out by appropriate laboratory tests. Although ketogenic diet can cause chronic bone demineralization resulting in chronic hypercalcemia,^11^ there are no published cases of any such effects acutely. The elevation of alkaline phosphatase was likely due to effects on the liver and was treated with addition of choline, which resulted in normalization of the alkaline phosphatase in a few weeks. It remains unclear if such a normalization would have happened without the addition of choline.

Finally, with patient 6, we show that acute changes in liver function tests can also happen in the positive direction with ketogenic diet, as the patient with chronic elevation in liver function tests (in a mixed pattern, with high aspartate aminotransferase, alanine aminotransferase, and γ-glutamyl-transferase with normal alkaline phosphatase) showed acute improvement in aspartate aminotransferase and alanine aminotransferase and subsequent normalization after starting ketogenic diet with choline.

Choline directly affects cholinergic neurotransmission and lipid transport from liver. Choline deficiency can result in hepatic, renal, pancreatic, memory, and growth disorders.^12^ Although concrete data about use in ketogenic diet is lacking, many centers use choline supplementation (with sunflower lecithin) for elevation of liver function tests on a chronic basis (communication from www.charliefoundation.org medical forum). Animal studies^13,14^ support the use of choline supplementation in ketogenic diet to limit hepatic mitochondrial dysfunction and fat accumulation. However, larger clinical studies are needed to conclusively suggest the use and dosing in humans.

Although these data show that liver function tests can show significant acute changes during initiation of ketogenic diet, irrespective of the presence of medications known to be hepatotoxic, it does not provide insight into the pathophysiology, drug interactions, or etiology. We also saw no clear differences between these 6 patients and the remaining 19 patients who did not show these changes, all of which had refractory epilepsy due to multiple etiologies, during initiation of ketogenic diet to explain this phenomenon. Hence, larger studies with pooled data or meta-analysis are needed to study this in further detail.

## Conclusion

We are the first to report acute changes in liver function tests during initiation of ketogenic diet in 6 patients, with both hepatocellular and cholestatic patterns, with and without the concurrent use of hepatotoxic medications. In most patients, ketogenic diet can be continued successfully by making changes to medications or addition of choline. Ketogenic diet can also result in acute improvement in chronically elevated liver function tests with initiation. Further research is needed to see if these abnormalities can be self-resolved without any intervention while on ketogenic diet or if supplementation of choline should be considered on a routine basis in these patients. Hence, monitoring of liver function tests is an important part of ketogenic diet initiation acutely.
